# Stem Cells Therapy for Spinal Cord Injury: An Overview of Clinical Trials

**DOI:** 10.3390/ijms21020659

**Published:** 2020-01-19

**Authors:** Serena Silvestro, Placido Bramanti, Oriana Trubiani, Emanuela Mazzon

**Affiliations:** 1 IRCCS Centro Neurolesi “Bonino-Pulejo”, Via Provinciale Palermo, Contrada Casazza, 98124 Messina, Italy; serena.silvestro@irccsme.it (S.S.); placido.bramanti@irccsme.it (P.B.); 2 Department of Medical, Oral and Biotechnological Sciences, University “G. d’Annunzio” Chieti-Pescara, 66100 Chieti, Italy; trubiani@unich.it

**Keywords:** stem cells, spinal cord injury, clinical trials

## Abstract

Spinal cord injury (SCI) is a traumatic lesion that causes disability with temporary or permanent sensory and/or motor deficits. The pharmacological approach still in use for the treatment of SCI involves the employment of corticosteroid drugs. However, SCI remains a very complex disorder that needs future studies to find effective pharmacological treatments. SCI actives a strong inflammatory response that induces a loss of neurons followed by a cascade of events that lead to further spinal cord damage. Many experimental studies demonstrate the therapeutic effect of stem cells in SCI due to their capacity to differentiate into neuronal cells and by releasing neurotrophic factors. Therefore, they appear to be a valid strategy to use in the field of regenerative medicine. The purpose of this paper is to provide an overview of clinical trials, recorded in clinical trial.gov during 2005–2019, aimed to evaluate the use of stem cell-based therapy in SCI. The results available thus far show the safety and efficacy of stem cell therapy in patients with SCI. However, future trials are needed to investigate the safety and efficacy of stem cell transplantation.

## 1. Introduction

Spinal Cord Injury (SCI) is a serious central nervous system disorder that was recognized as an important global health priority [1]. SCI is frequently caused by road accidents (38%), falls (>22%), violence (13.5%), sports injuries (9%), and other common traumatic events [2]. However, non-traumatic conditions, such as cancer, inflammation, infections, or spinal disc degeneration, could also induce SCI [3]. This deleterious condition leads to a serious effect in subjects affected by this disorder, damaging their mental state, labor ability, and social behavior [4]. The main clinical manifestations of SCI include alterations of somatic sensation, voluntary movement, urinary function, and systolic and diastolic functions. The primary injury is due to the first mechanical trauma caused by the local deformation of the spinal column, which induces the loss of neurons. After mechanical damage occurs, compression of the spinal cord leads to consequent neuronal degeneration and blood vessel damage [5]. In this context, the inflammatory state is an important factor in the pathogenesis of SCI leading to neuron and oligodendrocyte loss with consequent damage to nerve tissue [6]. The occurrences that follow the injury and characterize this phase are called “secondary damage”. The post-traumatic inflammatory reaction, the formation of free radicals, vascular ischemia, edema, apoptosis. or genetically programmed cell death are the causes of secondary damage [7]. The current pharmacological treatment of SCI involves the use of high-dose corticosteroids, administered within the first 8 h after the injury and continued for 1–2 days [8]. Stem cell therapy might be an efficient tool for the management of SCI. A few preclinical studies of SCI were to prove the strength of stem cell therapy to enhance motor capability [9]. Among stem cells, Mesenchymal Stem Cells (MSCs) appear to be the most interesting for SCI repair. MSCs are cells that show the ability to differentiate into different types of tissue such as cartilage, bone, fat, and nervous tissue [10]. MSCs show their aptitude to repair nervous tissue damage that occurs after the injury, thanks to their capacity to differentiate into neurons [11]. Therefore, MSCs, exhibiting immunomodulatory and anti-inflammatory properties, can release inflammatory cytokines that reduce neuronal loss, thus emerging as a hopeful therapeutic strategy for the management of SCI [12,13,14]. The purpose of this review is to offer a briefing on current clinical studies recorded on ClinicalTrial.gov. These studies aim to evaluate the efficacy and safety of the therapeutic application of different stem cells for the care of patients with SCI.

## 2. Spinal Cord Injury

SCI is a complicated pathology due to an important mechanical injury to the spinal tissue. This condition is responsible for the partial or total loss of the sensorimotor capacity, resulting in paraplegia or quadriplegia. This disease is characterized by three consecutive phases: acute, subacute, and chronic [15]. The first phase triggers an inflammatory response that leads to the infiltration of inflammatory elements such as macrophages, T lymphocytes, neutrophils, and microglia that release cytokines with pro-inflammation function, such as Tumor Necrosis Factor alpha (TNF-α), Interleukin 1-beta (IL-1β), and Interleukin-6 (IL-6) [16,17]. In addition to the inflammatory response, the formation of free radicals also plays an important role in secondary SCI damage. At the mitochondrial level, inadequate reduction of oxygen molecules causes the formation of excessive levels of Reactive Oxygen Species (ROS), responsible for various harmful events such as lipid peroxidation, DNA damage, and cell death [18]. The increase in ROS and Reactive Nitrogen Species (RNS) aggravates tissue loss. The peroxidation of free radicals and lipids causes oxidative death in neurons [19]. All these mechanisms are involved in the progressive damage of the tissue surrounding primary trauma, known as “secondary injury” [20]. These events are followed by a subacute phase, which in humans lasts about one year [15]. Several days after the lesion, the further expansion of the damage leads to chronic disease. Furthermore, the activation of astrocytes causes the formation of excessive levels of the extracellular matrix, which, together with an unfriendly environment and the increase of neuronal death, are responsible for the inhibition of cell migration and repair of axonal growth. In this way, large cystic cavities are formed at the site of the injury [21,22].

Following spinal trauma, it is important that patients have their spinal cord immobilized to reduce traumatic damage [23]. In traumatic SCI, early intervention, between 8 and 24 h after trauma, by surgical decompression is essential to minimize the extent of damage [24]. The decompression intervention later injury is fundamental to relieve compression of the spinal cord, thus reducing the area of the lesion and enhancing the vascularization in the injured site [25]. These above-mentioned occurrences demonstrate that SCI is a multifactorial condition that could lead to sensorimotor loss of limbs and trunk, as well as the loss of autonomous (involuntary) body adjustments. This complex pathology could be aggravated by other frequent dysfunction such as changes in respiratory and cardiac function, temperature control, blood pressure, bowel and bladder management, and sexual function [15].

Drug treatment in acute SCI appears to be an important strategy used to improve neurological function and promote nervous tissue repair. Several promising drugs have been tested, but few show the potential for application in patients with SCI. To date, Methylprednisolone Sodium Succinate (MPSS) is the first-line therapeutic option, administrated in the first hours after the injury with elevated dosage for 48 h to reduce severe edema development [26,27]. MPSS is a unique therapeutic agent validated by the Food and Drug Administration as a standard therapeutic tool in acute SCI management. MPSS is a synthetic glucocorticoid that can inhibit the lipid peroxidation, preserve the blood–spinal cord barrier, improve spinal cord blow flow, reduce the inflammatory response, and decrease oxidative state, consequently inducing an increased neural cell vitality [8]. However, to date, clinical studies have shown that no tested therapeutic option is efficacious in the treatment of patients with SCI.

Thus, several studies focus on identifying new pharmacological targets that could be promising for the management of SCI.

## 3. Spinal Cord Injury and Stem Cells therapy

SCI is a complex medical and living condition reflected in loss or impairment of functions that lead to reduced mobility and/or sensitivity [28]. Interventions to improve the lives of people with SCI in use today, including drug treatments, surgeries, and rehabilitation therapy, provide poor outcomes [29]. Therefore, it is important to find safe and efficacious treatments that can repair SCI, a goal that is still far away. The use of stem cells in the treatment of SCI has recently entered the clinic as a possible therapeutic application [30]. Stem cell transplantation is used in regenerative medicine to regenerate injured neurons. In several preclinical studies, conducted on animal models of SCI, stem cell therapy has promoted enhanced motor activity and neurological functions [31]. In particular, neuronal stem cells and MSCs, such as Bone Marrow MCSs (BM-MSCs), Adipose Tissue-derived MSCs (AT-MSCs) and Umbilical Cord MSCs (UC-MSCs), appear capable of regenerating damaged nerve tissue, as demonstrated in several in vivo studies, using experimental models of SCI [11,32].

Neuronal stem cells are multipotent cells isolated from the lateral ventricle, the dentate gyrus of the hippocampus, and the central canal of the spinal cord. These cells can differentiate towards neurons, oligodendrocytes, and astrocytes [33]. A preclinical study observed that the administration of neuronal progenitor cells showed good survival and differentiation capacity and promoted functional recovery [32]. Additionally, neuronal stem cells accelerated axonal growth at the injury area and modestly improved axonal conduction after stem cell administration [34]. Moreover, it was demonstrated that neuronal stem cells secrete numerous growth-promoting factors including Brain-Derived Neurotrophic Factor (BDNF), Glial Cell-Derived Neurotrophic Factor (GDNF), and Insulin Growth-Factors-1 (IGF-1) [35]. Neural stem cells, when administrated to an experimental model of thoracic spinal trauma, promoted the differentiation of oligodendrocytes and improved myelination, and thus motor and sensory function [36]. The therapeutic effects of neuronal stem cell transplantation also include immunomodulatory effects. Indeed, these cells show the ability to regulate T cells and macrophages in order to reduce inflammatory demyelination [37]. However, harvesting neuronal stem cells is limited by donor morbidity and their limited expansion.

AST-OPC1 is an experimental compound, defined as an “orphan drug”, candidate for the care of damage caused by acute SCI. AST-OPC1 (formerly GRNOPC1) is a cell therapy substance made up of Oligodendrocyte Progenitors Cells (OPCs) that was initially derived from the H1 human Embryonic Stem Cell (hESC) line [38]. In a preclinical experiment, AST-OPC1 was shown to possess several potentially beneficial capacities for the treatment of SCI. AST-OPC1 appears to be able to produce neurotrophic factors, stimulate vascularization, and induce remyelination of damaged axons [39].

MSCs gained attention because, in comparison to stem cells, they show several advantages. MSCs raise no ethical concern and show a limited risk of developing tumors [40]. Generally, MSCs are easily harvested and can be obtained from different types of tissues (including bone marrow, umbilical cord, adipose tissue, placenta, and oral cavity), do not show cytogenetic variation when expanded in culture for long periods, and can differentiate towards several lineages such as neuronal cells [10]. In compliance with the International Society for Cellular Therapy, MSCs own the minimum requisitions for being classified as stem cells, including fibroblast-like morphology and plastic-adherence when grown under standard terms. Moreover, MSCs positively express surface markers CD73, CD90, and CD105, as well as the adhesion molecules CD106 (vascular cell adhesion molecule), CD166 (activated leukocyte cell adhesion molecule), intercellular adhesion molecule-1, and CD29. Contrarily, they negatively express hematopoietic markers CD45, CD34, CD14, CD11b, CD79a, and CD19 as well as major histocompatibility complex class II surface molecules [41]. MSCs show neuroprotective effects in different neurodegenerative diseases [42,43]. They also exhibit immunomodulatory and anti-inflammatory roles in various immune system diseases and inflammatory disorders [44]. Indeed, MSCs, by suppressing the T and B cell proliferation rate and silencing the natural killer cells signaling, promote the regulation of immune response [14,45]. Moreover, MSCs can supply immunomodulatory effects and trophic support in SCI, by the delivery, at the damaged tissue site, of several growth factors including BDNF, GDNF, Vascular Endothelial Growth Factors (VEGF), and Nerve Growth Factor (NGF) [46]. Additionally, MSCs reduce apoptosis [13] and pro-inflammatory cytokines, such as TNF-α, IL-1β, IL-6, IL-4, IL-2, and IL-12 [47]. Therefore, MSCs prevent second-phase neuronal damage by inhibiting the inflammatory response in the lesion area after traumatic injury [48].

BM-MSCs harvested from the bone marrow can differentiate towards mesodermal cells. Several studies report the powerful capacity of BM-MSCs to differentiate into osteoblasts, chondrocytes, chondroblasts, adipocytes, fibroblasts, and various typologies of neurons and glial cells [49,50]. It was demonstrated that BM-MSCs induce repair of tissue damaged via activation of different mechanisms. Principally, BM-MSCs, by reducing the rates of proliferating and differentiating of lymphocytes, exhibited anti-inflammatory properties [51,52]. Furthermore, BM-MSCs provide trophic support and neuroprotection through the delivery of different growth factors including BDNF, VEGF, NGF, GDNF, Neurotrophin-3 (NT-3), Fibroblast Growth Factor (FGF), and Epidermal Growth Factor (EGF); thus, they appear able to repair the lesion area damaged from additional neuronal loss [53,54].

AT-MSCs are isolated from adipose tissue through liposuction and lipoplasty surgery. The ability of AT-MSCs to secrete many neurotrophic factors (e.g., BDNF and GDNF) [55,56,57], extracellular matrix substances, proteases, cytokines (e.g., IL-6, IL-7, IL-1, IL-8, and TNF-α), and immunomodulatory molecules (including IL-10 and Transforming Growth Factor-beta [TGF-β]), shows their promising neurodegenerative, anti-apoptotic, angiogenic, and wound healing effects [58]. In addition to these mechanisms of action, AT-MSCs can also differentiate towards several cell lines such as neuron cells, cells derived from endothelial, and Schwann cells [59]. AT-MSCs, thanks to their multi-differentiation properties, are involved in changing/enriching the lesion area of neuron cells damaged or undergoing necrosis; thus, they have the capacity to repair nerve tissue damaged in patients with SCI [58]. Different studies conducted in animal models of SCI and in humans have shown that AT-MSCs, when administered directly in the lesion area after injury, exerted a potential regenerative effect [57,60,61].

UC-MSCs are obtained easily by treating the umbilical cord or the cord blood from infants and can be stored at cryogenic temperatures until use. These cells show a low immunogenicity power and less risk of rejection after transplantation compared to other stem cells [62]. Their biological effects are mainly conferred by the release of cytokines and trophic factors, including IL-1, IL-10, neutrophil activator, NT-3, BDNF, VEGF, FGF, and neural cell adhesion molecule [9,63,64], which promote nervous tissue repair, induce axon growth, and activate damaged neurons. Much preclinical evidence demonstrate that UC-MSCs have broad curative effects [65] with a combination of multiple factors efficacy in several experimental models of SCI, such as neurotrophic [66], anti-inflammatory [63,66], anti-apoptotic [67], and angiogenic effects [68]. UM-MSCs are normally harvesting from a gelatinous matrix known as Wharton’s Jelly (WJ-MSCs) [69]. Some researchers observed that WJ-MSCs compared to BM-MSCs have a greater proliferative capacity; therefore, they can be propagated more quickly and extensively [70]. Moreover, in a preclinical study, it was observed that administering human WJ-MSCs induced the release of the trophic factors in the lesion area as well as axonal regeneration in situ. These results show that these cells enhance function motor [71].

Although stem cells represent an important therapeutic strategy, they still have several limitations. One of the major problems of stem cell transplantation is the potential risk for tumors and unforeseen alteration in phenotype. Although stem cell transplantation appears to be safe in the clinical trials performed to date, the risk of possible immune reactions should always be considered [72]. Another issue regarding stem cell transplantation is the choice of route of administration. The selection of a safe and efficacious route of administration is critical to the efficacy of the treatment. Indeed, some routes of administration such as the intramedullary route may be invasive, while other routes such as the intrathecal route may be less efficient because they require many cells [73].

A low rate of neuronal differentiation and a low survival rate are also problems that could occur after cell transplantation [74].

## 4. Stem Cells therapy in Spinal Cord Injury in Human

**Figure 1:**
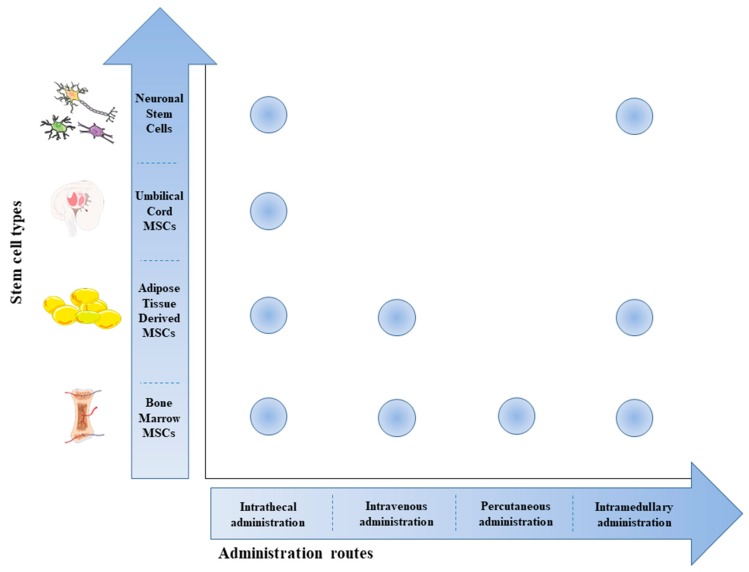
Scheme of stem cell types used in SCI treatment and their routes of administration. The most used route of administration is the intrathecal one, regardless of the type of cells used. The intramedullary route was used in five studies for the transplantation of Bone Marrow Mesenchymal Stem Cells (BM-MSCs), Adipose Tissue Mesenchymal Stem Cells (AT-MSCs), and Neuronal Stem Cells. Only three trials used the percutaneous route of administration for BM-MSC transplantation and two trials used the intravenous route for the transplantation of AT-MSCs and BM-MSCs. The image was created using the image bank of Servier Medical Art (available at http://smart.servier.com/), licensed under a Creative Commons Attribution 3.0 Unported License (https://creativecommons.org/licenses/by/3.0/).

In recent decades, several studies were performed to evaluate the effects of stem cell therapy as a therapeutic tool in SCI. This review offers a summary of the clinical trials registered on http://clinicaltrial.gov. We include all clinical trials (completed and still ongoing) that evaluate the effects of different stem cells transplanted via several routes of administration (Figure 1) in the management of SCI.

### 4.1. Completed Clinical Trials

All clinical trials (Phases 1, 2 and 3) described below aimed to show the safety and/or efficacy of stem cell use as a therapeutic tool in SCI (Table 1).

#### 4.1.1. Bone Marrow Mesenchymal Stem Cells

An open, Phase 1 study NCT02152657 aimed to evaluate the feasibility in terms of safety and efficacy of percutaneous administration of BM-MSCs in subjects with chronic SCI. In this study, five patients (aged 18–65 years) who were affected by complete paraplegia and SCI for at least six months classified as American Spinal Injury Association (ASIA) A were enrolled. After surgical procedures, the patients underwent a series of neurological evaluations. Additionally, several clinical exams was performed in all patients, including cell blood count, evaluation of biochemical parameters (dosage of electrolytes: sodium, potassium, and magnesium), urea and creatinine analysis to evaluate the renal functionality, coagulation markers, liver functionality, glucose and total cholesterol analysis to assess the metabolic profile, and urine and culture analysis. Serology needed for blood transfusion and marrow transplant, electrocardiogram, and radiological exams were also performed. After six months from transplantation, sensitivity and motor strength on the inferior limbs were assessed using AIS degree on the ASIA score and laboratory tests were performed such as somatosensory evoked potential, Computed Tomography (CT), and Magnetic Resonance Image (MRI) of the spinal cord at the thoracic and lumbar level. The urological function was also tested to evaluate urological improvements. Finally, with the aim monitor progress in sensorial mapping and improvement in neuropathic pain, clinical analyses of the patients and questionnaires were performed. The results of this study are not yet available.

The Phase 1 study NCT01325103 evaluated the safety and potential efficacy of autologous administration of BM-MSCs in SCI. For the study, fourteen patients (aged 18–65 years) affected by thoracic or lumbar traumatic SCI were enrolled (ASIA grade A). All patients were subjected to isolation of BM-MSCs. From the anterior and posterior iliac crest of all patients 60 mL of bone marrow cells were aspirated. Cell separation and culture procedures were performed. A clinic dose of BM-MSCs was obtained after one-month culture and BM-MSCs at passages 3–5 were harvested. Cells characterization through flow cytometric evaluation, differentiation assay, and G-band karyotype analysis was performed and their sterility was also evaluated. Patients underwent laminectomy and decompression of the spinal cord in the lesion area. Subsequently, the dura mater was opened and 5 × 10^6^ cells/cm^3^ were administrated. In the six months after transplantation, all patients underwent rehabilitation therapy five times a week for 4 h per day for the first two months and for 2 h per day in the following months, with the aim to increase the efficacy of transplantation. For at least six months, clinical and neurological standard analyses were evaluated. Following treatment, the neurological assessments showed variably enhanced sensitivity below the injury area. Improvements in sensitivity were also recorded three months after transplantation. In eight subjects, improvements in lower limb motor functions were observed. Seven patients presented sacral sparing and showed the change from complete to incomplete lesion, followed by improvement in Association Impairment Scale (AIS) score to grade B or C. During the treatment no patients had improvements in urological function and, at three and six months after BM-MSC administration, the nuclear MRI showed no alteration in hyperintense signals, increase of lesion, or presence of new gliosis areas. Three months post-treatment, three patients showed an improvement of neuropathic pain with a significant reduction of pharmacological therapy. Contrarily, in three patients, temporary intensification of neuropathic pain was recorded. Additionally, one subject presented a change in Somatosensory Evoked Potentials (SSEP). The outcomes of this clinical trial evidenced that the autologous administration of BM-MSCs is safe and feasible. Moreover, this demonstrated that the autologous cell therapy approach may be a valid tool that might induce motor function improvement and decrease the probability of developing secondary damage [75].

A non-randomized, Phase 1, interventional clinical trial (NCT02482194) aimed to assess the feasibility in terms of safety and efficacy of intrathecal delivery of autologous BM-MSCs in the management of SCI. Nine patients (aged 18–50 years) affected by thoracic complete SCI were included in this study. Depending on the time since the injury, the participants were separated into two groups. Group 1 included six participants with chronic SCI for at least thirty-three months, while Group 2 included the remaining three patients who had a subacute injury (<6 months). From the iliac crest of all patients, 50–60 mL of bone marrow aspirate were obtained and subsequently maintained in culture following the Good Manufacturing Practice. A clinical dose of BM-MSCs was obtained after one-month culture and BM-MSCs at passages 3–5 were harvested. Subsequently, all patients received 1.2 × 10^6^ cells/kg of body weight of BM-MSCs by intrathecal injection. Each patient received two or three courses of BM-MSCs on Day 1 and Weeks 4 and 8, respectively. All except one patient completed a one-year follow-up. During a median follow-up of 720 days in first group and 366 days in the second group, no severe adverse events were observed. After intrathecal injection, one patient experienced a headache, which resolved on the subsequent day. Two patients recounted non-specific tingling sensation. After one year, MRI did not detect any alteration in the hyperintense signal and no formation of ectopic tissue. In conclusion, all patients tolerated the procedure well during the one-year follow-up; therefore, autologous MSC transplantation by intrathecal injection can be considered a safe procedure. However, the absence of a control group and the short duration of patient monitoring are details that limit the conclusive results regarding the effectiveness of the transplant. These encouraging safety findings suggest that trials with placebo groups are necessary to evaluate the efficacy of BM-MScs [76].

Another clinical trial (NCT01909154) proposed to evaluate the feasibility in terms of safety and efficacy of local autologous BM-MSC transplantation in the lesion area. This Phase 1 study enrolled a cohort of twelve non-randomized patients (aged 18–60 years) with SCI (ASIA A). These patients showed complete paraplegia, which manifested a motor and sensory function loss below the injury area. Each patient was treated with autologous BM-MSC transplantation in the lesion area through intrathecal microinjection. Three months after the first surgery, all patients received a second dose by lumbar subarachnoid transplantation. Based on intramedullary post-traumatic injury, BM-MSCs were administrated at a minimum dose of 100 × 10^6^, while, after three months, 30 × 10^6^ BM-MSCs were transplanted via lumbar subarachnoid injections. All subjects underwent follow-up 12 months after the surgical intervention. After transplantation, the occurrence of adverse events was monitored by clinical evaluation every day during the first week and weekly until the six-month follow-up visit and then at Months 9 and 12. Moreover, efficacy-sensitivity recovery using ASIA scale and efficacy-changes in the level of chronic pain (based on the International Association of Neurorestoratology Spinal Cord Injury Functional Rating Scale (IANR-SCIFRS)) were evaluated and efficacy-changes in the neurophysiological parameters and efficacy-urodynamic were also assessed. The safety results show that no serious adverse events were recorded, while adverse events were recorded in each patient (such as nausea, urinary tract infection, back pain, thoracic pain, muscle contracture, myalgia, and headache). The efficacy results show that after 12 months patients had significant sensitivity recovery and an improvement in the level of chronic pain was observed. Antecedent to transplantation surgery, no SSEPs were recorded in the participants, while, 12 months after transplantation, seven patients showed the presence of SSEPs. Urodynamic studies were evidence of a decrease in detrusor pressure that is considered a clinical improvement. Moreover, the outcomes of this clinical trial demonstrated that the transplantation of autologous BM-MSCs is safe and efficacious as a therapeutic tool in SCI.

A Phase 1 clinical trial (NCT01328860) aimed to determine the safety of autologous Bone Marrow Progenitor Cells (BMPCs) administration in children who suffered SCI. AAnother aim of the trial was to evaluate if the late functional outcome was improved following transplantation using pre-transplantation spinal cord function as the control. This study included ten children (aged 1–15 years) with SCI classified as ASIA grade from A to D. All children received approximately 5 mL/kg of BMPCs under anesthesia. Subsequently intravenous BMPC injections were monitored for 24 h for any adverse events. In addition, evaluation on Days 1, 30, and 180 was carried out to verify the recovery of the motor and sensory score. The results of this study are not yet available.

A 1/2 phase clinical study NCT01186679 aimed to assess the safety and efficacy of autologous transplanted BM-MSCs. Twelve Indian subjects (aged 20–55 years) with complete spinal injury at the level between C4 and T12 were recruited. Specifically, eight patients with chronic SCI were infused with BM-MSC directly into the lesion site by performing glial scar resection. In four patients with acute and subacute injury, autologous BM-MSC transplantation was performed by intrathecal injection. The safety of BM-MSC autologous transplantation was assessed by observing adverse events and the efficacy of autologous transplantation using the ASIA compromise scale. The results of this study are not yet available.

In an NCT0816803 1/2 phase, blinded clinical trial, the efficacy of autologous BM-MSC transplantation combined with physical therapy was assessed. Seventy (10–36 years) chronic (SCI for more than 12 months) patients with thoracic spinal trauma were recruited in this trial. Participants were randomly assigned into two different experimental groups: in 50 participants, autologous BM-MSCs (cell therapy group) were administrated, while, in 20 participants, the standard rehabilitative therapy was performed (control group). Both groups were provided with physiotherapy programs for at least 1–2 h three times per week. Physiotherapy included mat activities, strengthening exercises, self-range of motion, ambulation training for paraplegic patients, and cardiopulmonary training. In all participants, rehabilitation therapy was stopped 2–3 days before receiving the transplant. Fifty participants received autologous BM-MSCs via intrathecal by lumbar punctures. In all participants, after transplantation, clinical and neurological (using the ASIA scale), SSEP, MRI, and functional independence measurements were performed. In the cell therapy group, moderate adverse effects such as headaches and mild pain that are usually common after lumbar punctures appeared in several participants. These temporary side effects disappeared spontaneously. The outcomes of this trial showed that 17 of the 50 subjects treated with intrathecal injections achieved ASIA conversion. Specifically, 2 of 15 participants who at baseline follow-up showed ASIA A SCI, changed to ASIA C and 6 changed to ASIA B. Moreover, after 18 months of follow-up, in 50 participants who received BM-MSC transplantation, 23 experienced an improvement of motor function. Patients treated with autologous BM-MSCs noticed a modest improvement in neurological functions 4–6 weeks post-transplant. Before therapy, at a baseline follow-up, no subjects had manifested cortical SSEP, while 65% of participants who received BM-MSC transplantation after one year experienced cortical impulses and nerve conduction enhancement. RMI showed an improvement of spinal cord compression and a reduction of in two participants at one year post-transplant. One year after the transplant, both these participants started walking again. These results demonstrate that autologous intrathecal injection of BM-MSCs is an efficacious therapeutic intervention that offers hope for all those people who suffer from SCI. In addition, the outcomes of this trial demonstrate that, for at least 18 months after the surgical procedure, transplantation is safe [77].

The potential clinical efficacy of intrathecal administration of autologous BM-MSCs was evaluated in another clinical trial (NCT02570932). This is a non-randomized Phase 2 study that enrolled a cohort of ten patients (aged 18–70 years) with established chronic SCI (A, B, C or D ASIA grade). Neurophysiology, MRI, and urology analyses for each patient were conducted before and after transplantation, with the aim to compare them to provide more reliable results on the effectiveness of the treatment. BM-MSCs were administrated via intrathecal in subarachnoid area through lumbar puncture. The total dose for each patient was 300 × 10^6^ cells, divided into three injections of 100 × 10^6^ cells. The day after transplantation was considered Day 1 of the experiment. After the first dose was administered, the other two cell doses were transplanted in Months 4 and 7. After the first cell transplantation, all patients had at least 10 months of follow-up. At the end of the study, motor and sensory function evaluations using the IANR-SCIFRS score were performed, with the aim to demonstrate the efficacy of stem therapy. Efficacy evaluation of transplantation was also assessed through the change in motor and sensory functions evaluation, employing the Penn Spasm Frequency Scale, and pain measurements, employing the Visual Analog Scale (VAS). Neurotrophic factors levels in cerebrospinal fluid were also collected. Additionally, to investigate the safety of transplantation, side effects were monitored by clinical analysis during the period treatment. The results of this study are not yet available. 

#### 4.1.2. Bone Marrow Mesenchymal Stem Cells versus Adipose Tissue-Derived Mesenchymal Stem Cells

The Phase 1/2 clinical trial NCT02981576 proposed to evaluate and relate the safety and efficacy of autologous transplantation of BM-MSCs versus AT-MSCs in subjects with SCI. In the study, fourteen patients (aged 18–70 years) with complete or incomplete SCI grade ASIA A, B, or C were enlisted. The patients recruited were blindly divided into two groups of equal numbers. Patients in one group received autologous BM-MSCs, while patients in the other group received treated with autologous AT-MSCs. Before transplantation from each patient, AT-MSCs or BM-MSCs were collected and processed in the laboratory. Afterwards, patients received AT-MSCs and BM-MSCs by intrathecal injections that were performed three times. A 12-month follow-up was accomplished to assess the safety of autologous BM-MSC transplantation compared to autologous AT-MSC transplantation. Moreover, the efficacy of autologous BM-MSC transplantation compared to AT-MSCs was evaluated by MRI using the ASIA scoring system. The results of this study are not yet available.

#### 4.1.3. Adipose Tissue-Derived Mesenchymal Stem Cells

The safety of autologous AT-MSCs transplants in patients with SCI was evaluated in another clinical trial (NCT01274975). This Phase 1 study was authorized by the Korea Food and Drug Administration with Investigational New Drug Application number of BPD-455 (29 April 2009) and the Institutional Review Board (No. 2009002-RNL-ASTROSTEM) at SAM Anyang Hospital (Anyang, Korea). In this study, eight male patients (aged 19–60 years) with traumatic SCI at least one year previously and classified as ASIA grade A or B were recruited. Before the collection of cells hematologic analysis was carried out, as well as the evaluation of urinary function. Patients were also checked for HIV, HBV/HCV, and VDR infections. Moreover, for each patient chest X-ray, pulmonary function measurements, spinal cord independence analysis, visual evaluation, spinal MRI, the electrophysiological test of motor and somatosensory evoked potentials, and neurological evaluation using ASIA were also obtained. AT-MSCs were isolated in sterile states and stored at high temperatures without being exposed to cryotemperature. Before transplantation, cell viability tests and the search for contaminants (contamination by fungi, bacteria, endotoxin, and mycoplasma) were also performed in the collected AT-MSCs. In each participant, an autologous AT-MSC transplantation at a dose of 4 × 10^8^ was infused intravenously for 3–4 h. After treatment, all subjects had follow-up visits on Days 1, 4, and 7 and one month and one year later. The outcomes of this study showed that no severe side events related to the surgery procedure appeared until the end of the trial. Several side events frequent in subjects who suffer from SCI were experienced in some participants. All side events were temporary and were resolved or stabilized; only one patient experienced an idiopathic form without symptoms of hyperthyroidism that did not resolve during the follow-up period. Three months after cell transplantation, no significant differences were observed in heart function, physical condition, or vital signs, compared to baseline. However, at 12 weeks post-transplantation, RMI revealed a non-significant reduction (*p* = 0.8047) of the spinal section lesioned. Additionally, one participant experienced an improvement in the disease by changing the degree of impairment from ASIA A to C, with a six-point improvement in the motor function as well as improved sensory function (20 points for the sensory puncture and two points for a light sensory touch). The outcomes of this study are encouraging; however, the lack of control group, the restricted follow-up duration, and the inadequate number of participants do not allow obtaining significant statistical data to establish the efficacy and safety of the autologous administration of AT-MSCs [78].

Another Phase 1clinical trial (NCT01624779) evaluated the efficacy of intrathecal transplantation of autologous AT-MSCs. In this study, fifteen patients (aged 19–70 years) with SCI were enrolled. AT-MSCs was collected from each patient and subsequently cultured. Clinical doses of 9 × 10^7^ cells/3 mL were administrated via intrathecal injection at Day 1, Month 1, and Month 2. To test the efficacy of transplantation, MRI change was evaluated six months after intervention and compared to MRI before transplantation. Changes in neurologic and electrophysiological function were also observed before and after intervention. Additionally, adverse events were recorded to assess the safety of autologous transplantation of AT-MSCs by intrathecal injection in subjects with SCI. This trial was completed; however, the results are not yet available.

The feasibility in terms of safety and efficacy of autologous AT-MSC administration was investigated in another clinical trial (NCT01769872). For the study, fifteen patients (aged 19–70 years) with SCI (ASIA grade A, B or C) were enrolled. In each patient, AT-MSCs were administrated via multiple routes and at different doses: intravenous at a dose of 2 × 10^8^ cells/20 mL, intrathecal at a dose of 5 × 10^7^ cells/2 mL, and directly into the spinal cord at a dose of 2 × 10^7^ cells/1 mL. Changes in the spinal cord compared to baseline were observed three and six months after the administration of AT-MSCs to assess the efficacy of the transplant. Moreover, the MRI was performed three and six months after treatment. The presence of side events was also recorded to evaluate the safety of autologous transplantation of AT-MSCs for SCI treatment. The results of this study are not yet available.

#### 4.1.4. Umbilical Cord Mesenchymal Stem Cells

The Phase 2 clinical trial NCT01393977 aimed to evaluate the efficacy of UC-MSC transplantation in relation to rehabilitation therapy and self-healing in patients with thoracolumbar SCI. The study was authorized by the Ethics Committee of the General Hospital of Chinese People’s Armed Police Forces. For the study, thirty-four patients (aged 20–50 years) with thoracolumbar SCI classified as ASIA grade A were enrolled. The patients were randomly assigned into three groups: the stem cell transplantation group, the rehabilitation therapy group, and the control group. Patients in the first experimental group received a total of 4 × 10^7^ UC-MSCs directly into subarachnoid through lumbar puncture. Participants in the second experimental group followed a rehabilitative procedure for 90 days, such as training of motor and urinary function. The third group of participants did not receive any intervention. All participants underwent baseline follow-up and six months after the start of the study. Muscle potency, self-care capacity, muscle tension, and limb sensitivity were assessed using the ASIA score, the Barthel Index, and the manual muscle strength and muscle tension scale. After treatment, no side effects occurred; however, one participant experienced radiating neuralgia after UC-MSC transplantation that was resolved 24 h later. Six months after treatments, seven of the ten participants who received UC-MSCs showed a significant enhancement of motor function muscular tension and self-care capacity. Instead, five of the fourteen subjects who received the rehabilitation interventions showed an enhanced of this function but without significant statistical evidence. Contrary to patients undergoing physiotherapy, subjects treated with UC-MSCs showed improved urinary function. In particular, urodynamic analyses showed an improvement in the maximum urinary flow rate and maximum bladder capacity, as well as a reduction in the volume of residual urine and the maximum detrusor pressure. In conclusion, the patients treated with UC-MSC transplantation showed a significant enhancement of motor function (such as improvement in limb muscle tension and increased limb strength) and urinary function compared to patients who received rehabilitation therapy or without treatment. These findings demonstrate that UC-MSC administration improves the quality of life in subjects affected by SCI and that this treatment is more efficacious than rehabilitation therapy and self-healing [79]. A few years later, the same research team started an identical Phase 3 clinical trial (NCT01873547). This study enrolled 300 patients (aged 20–65 years) with SCI for at least one year. First, 300 patients were divided into three groups: 100 subjects were treated with UM-MSCs via intrathecal injection; 100 patients only received rehabilitation for 14 months; and 100 participants received neither treatment. In all patients before treatment and six and twelve months later, clinical and neurological evaluation using the ASIA scale, electrophysiological SSEP, and motor evoked potentials were performed, with the aim to assess the efficacy of treatments. The findings of this trial are not yet available.

#### 4.1.5. Neuronal Stem Cells

A Phase 1/2 NCT01321333 aimed to evaluate the feasibility in terms of safety and efficacy of intramedullary administration of human CNS Stem Cells (CNS-SCs) in patients with thoracic (T2–T11) SCI. The study enrolled twelve patients (aged 18–60 years) with thoracic sub-acute SCI classified as ASIA grade A, B or C and with T2–T11 thoracic lesions assessed by MRI and/or Computerized Tomography (CT). Single dose of human CNS-SC cells was administrated via intramedullary injection in each patient. Post-treatment participants were treated with immunosuppressant for nine months after transplantation and all were monitored for one year. At the conclusion of this trial, the patients were recruited in another trial with a follow-up of four years (NCT01725880). The investigation is an observational study that aimed to monitor the patients to assess the safety and efficacy of CNS-SC transplantation in the long term. The results of these studies are not yet available.

A similar single-blind, randomized, Phase 2 clinical trial (NCT02163876) was conducted a few years later. The study enrolled thirty-one participants (aged 18–60 years) with cervical SCI classified as ASIA grade B or C and with a lesion at C5–C7 motor levels. The enrolled subjects were divided into two arms: the experimental arm and the no-intervention arm. The patients of the experimental group were subjected to intramedullary transplantation of human CNS-SCs in the cervical spine. The subjects of the no intervention group did not undergo any surgical treatment and represented the control group. Up to one year after enrollment, change from baseline in upper extremity motor scores was evaluated using the International Standards for Neurological Classification of SCI to test the efficacy of transplantation. The secondary purpose of the trial was to evaluate the safety of human CNS-SC transplantation. In this context, the number of participants with serious and non-serious adverse events was collected. Additionally, laboratory tests, neurological examination, pain and allodynia assessment, and physical examination were also performed. The results of this study are not yet available.

An open-label clinical trial NCT02302157 aimed to evaluate the feasibility in terms of safety AST-OPC1 transplantation in subjects with subacute cervical SCI. For the study, 25 patients (aged 18–65 years) with sensorimotor complete traumatic SCI were enrolled. These patients were divided into five cohorts. Cohorts 1–3 showed a traumatic SCI with ASIA impairment Scale A, while Cohorts 4 and 5 showed ASIA impairment scale B. Patients received increasing doses of AST-OPC1 at a specific time between 21 and 42 days after the injury. Patients were randomized to five cohorts that received one administration of 2 × 10^6^ or 10 × 10^6^AST-OPC1 cells or two administrations of 10 × 10^6^AST-OPC1 cells for a total of 20 × 10^6^ cells. At the end of the experiment the number of adverse events within one year related to AST-OPC1 injection was recorded. Moreover, neurological function level using ISNCSCI was also assessed. The results of this trial are not yet available.

The findings of this terminated trial demonstrate that stem cell therapy is safe and efficacious. However, many studies still lack results that will be needed to encourage stem cell therapy as therapeutic tool in SCI treatment.

### 4.2. Ongoing Clinical Trials

Over the past 10 years, various trials have been promoted to evaluate the efficacy and safety of stem cell therapy in patients with SCI. In this section, we summarize all clinical studies that are still undergoing patient recruitment (Table 2).

#### 4.2.1. Umbilical Cord Mesenchymal Stem Cells

Three Phase 2 clinical trials (NCT03505034, NCT03521336, and NCT03521323) aim to assess the feasibility in terms of safety and efficacy of intrathecal administration of allogeneic UC-MSCs for the treatment of different phases of SCI. The purpose of these three studies is to evaluate the efficacy of UC-MSC administration to find the best time for transplantation. In NCT03505034, participants with late-stage chronic SCI will receive UC-MSC transplantation and it is expecting to recruit about thirty subjects (aged 18–65 years). NCT03521336 will treat subjects with sub-acute SCI and is expecting to recruit about eighty-four subjects (aged 18–65 years). NCT03521323 will treat patients with early-stage of chronic SCI and is expecting to recruit about sixty-six individuals (aged ≥18 years). These studies will include two experimental groups. Participants in the experimental arm will receive intrathecal administration of UC-MSCs, at a dose of 1 × 10^6^ cells/kg, once a month for four months, while patients in the control group will receive 10 mL saline, once a month for four months. In all three studies, the recruited subjects will be evaluated before treatment and every three months for a maximum of one year post UC-MSC administration. From each patient, serum and cerebrospinal fluid will be harvested, at baseline and after transplantation, with the aim to understand the efficacy of UC-MSC transplantation as therapy for SCI. For all three clinical study, the last data reporter and the outcomes will be available by the end of 2021.

NCT03003364 is a Phase 1/2a, randomized, double-blind study that aims to evaluate the feasibility regarding both safety and efficacy of intrathecal injection (L3 level) of expanded WJ-MSCs. Ten patients (aged 18–65 years) affected by chronic traumatic SCI with complete paraplegia (ASIA A) will take part in the study. Ex vivo cultured human WJ-MSCs will be administered to patients in the experimental group by intrathecal injection. Following the administration, patients will remain for 24 h at the hospital and thereafter will be discharged. For the first period, the follow-up is planned on Day 7 and at 1, 3, and 6 months. At Month 6, the patients assigned to the placebo group will be treated in a crossover way (second period) and will follow the same schedule for the follow-up. The first clinical trial evaluation will be performed at the 12-month follow-up. To assess the safety and efficacy of the second dose of cells, at 12 months after the first infusion, patients will be randomized again to active treatment or placebo (double-blind). At 18 months after the time of inclusion, the patients assigned to the placebo group will also receive the second dose of WJ-MSCs Thereafter, patients at 24 and 36 months will be analyzed as part of the long-term follow-up. For this clinical trial, the findings are still unpublished.

#### 4.2.2. Adipose Tissue-Derived Mesenchymal Stem Cells

An open-label, prospective Phase 1 study (NCT03308565) will evaluate the safety and feasibility of the cerebrospinal fluid AT-MSC transplantation in ten subjects with SCI of grade ASIA A or B (aged ≥18 years). Recruited participants will undergo surgery, in which a small portion of the adipose tissue will be collected through a small incision in the abdomen or thigh. The sample taken will be used to obtain AT-MSCs, which will be kept in culture for 4–6 weeks. A single dose of 100 million autologous AD-MSCs will be transplanted by L4–L5 intrathecal injection under fluoroscopic guidance. After treatment, all participants will be assessed at Days 2 and 3 and at Weeks 1, 2, 4, 24, 48, 72, and 96 to assess the incidence of adverse events. The outcomes of clinical trial (estimated last reporter harvesting in November 2023) will probably clarify whether AD-MSC administration via the cerebrospinal fluid is safe.

#### 4.2.3. Bone Marrow Mesenchymal Stem Cells

Two clinical trials (NCT02574572 and NCT02574585) will study the safety and efficacy of autologous BM-MSC transplantation as treatment for SCI. NCT02574572 is a pilot, Phase 1 clinical trial in a prospective cohort. The population enrolled for the trial will be composed of ten participants (aged 18–65 years) who have had cervical and complete SCI for at least one year (grade ASIA A). All subjects will be subjected to laminectomy and autologous BM-MSCs intralesional administration. Instead, NCT02574585 is a randomized, non-placebo-controlled Phase 2 clinical study. Forty subjects will be enrolled (aged 18–65 years) who have had thoracolumbar chronic and complete SCI for at least one year (ASIA grade A). The patients will be randomly divided into groups, twenty of whom will receive two percutaneous injections of BM-MSCs, with a three-month interval between the injections. The other twenty patients will not receive any specific intervention and they will be clinically followed. To evaluate the safety of BM-MSC transplantation, in both trials, the number of patients with side events associated with transplantation will be observed by MRI. Functional improvement in ASIA grade after 12 months of treatment will also be evaluated. Additionally, the subjects will receive questionnaires and will undergo to clinical analysis with the aim to verify enhancements in sensorial mapping and neuropathic pain. These trials are still ongoing and the data are unpublished.

The Phase 2/3 study NCT01676441 will assess the safety and efficacy of autologous BM-MSCs transplanted directly into the SCI lesion. The trial is expecting to recruit about thirty-two patients with traumatic SCI at the level of cervical with ASIA grade B. After laminectomy, in all subjects, autologous BM-MSCs will be injected into the intramedullary space at a dose of 1.6 × 10^7^ cells and in intrathecal space at a dose of 3.2 × 10^7^ cells. After recovery from the surgical intervention, the patients will undergo one month of physical and occupational treatment. The researchers will evaluate the modification in motor and sensory grade of compromising of the ASIA scale compared baseline and after 1, 3, 6, and 12 months of BM-MSCs treatment. This trial is still active and the data will be collected at the end of 2020.

The double-armed, Phase 1/2 clinical trial NCT02687672 will compare bone marrow and leukapheresis as sources to obtain autologous CD34^+^ and CD133^+^ stem cells, to be used as therapy for subjects affected by chronic complete SCI. This trial is expecting to recruit about fifty children and adults (aged 5–50 years) with chronic SCI. Enrolled subjects will be divided into two experimental cohorts. The first cohort of patients will receive the injections of autologous leukapheresis-derived stem cells, while the other group of patients will be treated with the injections of BM-MSCs. The first outcome of this clinical trial will assess improvements of sensory and motor function by ASIA at five-year follow-up. No other information or data are available yet.

#### 4.2.4. Neuronal Stem Cells

Phase 1, open-label, the clinical trial NCT01772810 will aim to assess the safety of human spinal cord-derived neural stem cells as therapy for chronic SCI. This study is still under recruitment; to date, eight subjects (aged 18–65 years) have been recruited and were assigned into different arms. Group A recruited four patients with a SCI at T2–T12 levels and Group B recruited four patients with a C5–C7 SCI. The clinical trial duration will be six months post-operation, during which adverse events and clinically significant laboratory abnormalities will be recorded. After treatments, patients will be followed for 54 months. To date, no information is available yet and the final data collection is expected by the end of 2022.

The Phase 1 study NCT04205019, not yet under recruitment, aims to assess the safety of “Neuro-Cells” in subjects affected by SCI. Neuro-Cells is a fresh low immunogenic human bone marrow-derived stem cell preparation prepares according to the manufacturing standard operating procedures of Neuroplast BV (Maastricht, Netherlands). The trial will enroll ten patients (aged 18–40 years) with traumatic complete (ASIA grade A) and incomplete (ASIA grade B/C) SCI (level of injury at C5–T12). The BM harvesting will be performed at the start of trial for each patient and after they will receive one dose of Neuro-Cells by intrathecal administration. To evaluate possible adverse events, all participants will be subjected to follow-up every month during this three-month study, by physical examination and biochemical analysis of their blood/urine. On Days 0 and 90, patients will also undergo a comprehensive neurological examination to evaluate the possible improvement in neurological condition and pain perception. Finally, participants will also be invited to undergo neurological examinations on Days 360 and 720. The purpose of this neurological assessment is to explore in patients if a late administration of Neuro-Cells can show positive effects on the neurological condition of the chronic SCI. This trial has not yet started and the final data collection is expected in March 2022.

A double-blind, placebo-controlled Phase 2/3 clinical trial (NCT03935724) will establish the safety and efficacy of the administration of Neuro-Cells in humans for treating SCI. Enrollment in Phase 3 of the study will automatically start once all patients have enrolled and been treated in Phase 2. Phase 2 starts with the enrollment of 16 patients with sub-acute SCI (6–8 weeks after the traumatic incident), randomly divided into two groups of eight patients each: 1A Intervention group and 1A placebo group. After an intervention analysis, the Phase 2 study will expand into a Phase 3 study, requiring the recruitment of 54 additional patients, who, subsequently, will be randomly divided into two groups of 27 patients each: 2A Intervention group and 2A placebo group. Both the Phase 2 and Phase 3 parts of the trial have a similar setup, which includes a screening period (1–2 days), randomization, a treatment period of one or two days, and a total follow-up period of one year. All patients will undergo a bone marrow harvesting at the start of their participation in the study. Approximately six months after the time of inclusion, in both phases, the patients assigned to the placebo group will undergo a second bone marrow harvesting and will also receive Neuro-Cells. All subjects will be treated with Neuro-Cells and complete follow-up for at least six months post-treatment. The trial is completed when the last patient finishes his/her last visit, approximately one year after the time of inclusion. For this study, the last data recording will be available in January 2021 and will make it possible to clarify the feasibility regarding the safety and efficacy of human autologous Neuro-Cells administration. 

## 5. Conclusions

SCI is a complicated disease that progresses over time, producing a worsening of the injured nerve tissue that exacerbates clinical outcome. There are no efficacious and safe treatments available in the management of this pathology. Stem cell transplantation represents a hope in the field of regenerative medicine that aims to find a cure for the devastating disease of SCI. Several in vivo studies and humans trials demonstrated that the use of stem cells as a therapeutic tool induces an improvement of motor function and neurological condition. The administration route for transplantation of stem cells is essential for the efficacy of this therapeutic intervention. Intravenous, intrathecal, and intramedullary are the most frequent routes of stem cell transplantation. All these routes of administration are considered safe; however, intravenous and intrathecal injections are less invasive then intramedullary administration. The transplanted cell doses are different according to the chosen route of administration and the phase of SCI. Specifically, several completed studies showed that BM-MSC administration into SCI subjects improved motor function and quality of life for at least one year. A limit of these studies is the small number of participants. Thus, investigations with more subjects are required. However, these studies are important to evaluate the safety and feasibility of the clinical use of BM-MSCs given that no severe side effects were recorded. Regarding efficacy, safety, and feasibility of AT-MSCs and UC-MSCs, the findings of clinical trials showed a limited understanding. Even though slightly improved sensory function was observed, longitudinal studies with important positive effects in motor function are still missing. It seems clear that both AT-MSCs and UC-MSCs need more clinical data. The expected results and future research are needed to optimize the employment of cell therapy in patients with SCI. Additionally, ongoing trials will be useful for researchers to clarify clinical use of stem cells as therapy for SCI.

## Figures and Tables

**Table 1:** Completed clinical trials of stem cell therapy in SCI (https://clinicaltrials.gov/). The table shows the efficacy and safety of stem cell therapy in the management of SCI.

Identifier	Study Title	Phase	Subjects	Cell Therapy	Route of Administration	Intervention	Efficacy	Security	Ref
Bone Marrow Mesenchymal Stem Cells									
NCT02152657	Evaluation of Autologous Mesenchymal Stem Cell Transplantation in Chronic Spinal Cord Injury: A Pilot Study	Phase 1	5 patients (18–65 years)	BM-MSCs	Percutaneous injection.	MSC transplantation	-	-	-
NCT01325103	Autologous Bone Marrow Stem Cell Transplantation in Patients With Spinal Cord Injury	Phase 1	14 patients (18–65 years)	BM-MSCs(5 × 10^6^ cells/cm^3^; single dose)	Intralesional injection	Autologous BM-MSC transplantation	Variable improvements in sensitivity were recorded in all patients and eight of these developed in lower limb motor functions. Seven patients improved AIS. Three subjects ameliorated the neuropathic pain and one presented changes in SSEP.	All patients were discharged within 48 h after surgery. One subject developed complication of cerebrospinal fluid leak, not related to the transplantation but rather to the intervention practices. No patients presented severe side effects or other complications.	[75]
NCT02482194	Autologous Mesenchymal Stem Cells Transplantation for Spinal Cord Injury- A Phase I Clinical Study	Phase 1	9 patients (18–50 years)	BM-MSCs (1.2 × 10^6^ cells/kg; two or three dose)	Intrathecal injection	Autologous BM-MSC transplantation	After 1 year of treatment, no participant in the MRI analysis showed a change in the hyperintense signal or presence of ectopic tissue.	No severe side events were recorded in any subjects. One patient complained of severe headaches, while two patients accounted for the non-specific tingling sensation.	[76]
NCT01909154	Safety Study of Local Administration of Autologous Bone Marrow Stromal Cells in Chronic Paraplegia	Phase 1	9 patients (18–50 years)	BM-MSCs (a minimum dose of 100 × 10^6^ and a second dose of 30 × 10^6^ after 3 months)	Intrathecal injection	Autologous BM-MSC transplantation (two doses)	After 12 months, patients had significant sensitivity recovery and an improvement in the level of chronic pain was observed. Seven patients showed the presence of SSEPs and improvement of urodynamic function was recorded.	No serious adverse events were recorded, while adverse events were recorded in each patient.	-
NCT01328860	Autologous Stem Cells for Spinal Cord Injury (SCI) in Children	Phase 1	10 children (1–15 years)	BMPCs	Intravenous infusion	Autologous BMPC transplantation	-	-	-
NCT01186679	Safety and Efficacy of Autologous Bone Marrow Stem Cells in Treating Spinal Cord Injury	Phase 1/2	12 patients (20–55 years)	BM-MSCs	Intrathecal injection	Autologous BM-MSC transplantation	-	-	-
NCT00816803	Cell Transplant in Spinal Cord Injury Patients	Phase 1/2	70 patients (10–36 years)	BM-MSCs	Intrathecal injection	Autologous BM-MSC transplantation	Seventeen of 50 patients treated with BM-MSCs showed achieved ASIA conversion and 17 patients observed an improvement in motor functions. Twelve months later, two patients recorded an improvement of nervous tissue damaged.	Most of the side effects were common with treatment was observed including headache and mild pain, buts all were transitory and totally solved.	[77]
NCT02570932	Administration of Expanded Autologous Adult Bone Marrow Mesenchymal Cells in Established Chronic Spinal Cord Injuries	Phase 2	10 patients (18–70 years)	BM-MSCs (three doses of 100 × 10^6^ cells)	Intrathecal injection	Autologous BM-MSC transplantation	-	-	-
	**Bone Marrow Mesenchymal Stem Cells versus Adipose Tissue-derived Mesenchymal Stem Cells**								
NCT02981576	Safety and Effectiveness of BM-MSC vs. AT-MSC in the Treatment of SCI Patients.	Phase 1/2	14 patients (18–70 years)	BM-MSCs and AT-MSCs (three doses)	Intrathecal injection	Autologous BM-MSCs or AT-MSC transplantation	-	-	-
**Adipose Tissue-derived Mesenchymal Stem Cells**									
NCT01274975	Autologous Adipose Derived MSCs Transplantation in Patient With Spinal Cord Injury.	Phase 1	8 patients (19–60 years)	AT-MSCs (4 × 10^8^ cells; a single dose)	Intravenous infusion	Autologous AT-MSC Transplantation	MRI, 12 weeks after administration of AT-MSCs, showed a reduction of the spinal section injured, but without significant difference (*p* = 0.8047). In one patient was observed conversion ASIA A scale in ASIA C and he showed an improvement in motor function and sensory functions.	No severe side effects associated with the intravenous administration were recorded. Nineteen adverse events were observed, but all resolved or stabilized during follow-up.	[78]
NCT01624779	Intrathecal Transplantation Of Autologous Adipose Tissue Derived MSC in the Patients With Spinal Cord Injury	Phase 1	15 patients (19–70 years)	AT-MSCs (9 × 10^7^ cells/3 mL; three doses)	Intrathecal injection	Autologous AT-MSC Transplantation	-	-	-
NCT01769872	Safety and Effect of Adipose Tissue Derived Mesenchymal Stem Cell Implantation in Patients With Spinal Cord Injury	Phase 1/2	15 patients (19–70 years)	AT-MSCs (2 × 10^8^ cells/20 mL, 5 × 10^7^ cells/2 mL and 2 × 10^7^ cells/1 mL cells)	Intravenous, Intrathecal and Intralesional injections	Autologous AT-MSC Transplantation	-	-	-
**Umbilical Cord Mesenchymal Stem Cells**									
NCT01393977	Difference Between Rehabilitation Therapy and Stem Cells Transplantation in Patients With Spinal Cord Injury in China	Phase 3	34 patients (20–50 years)	UC-MSCs (4 × 10^7^ cells)	Intrathecal injection	UC-MSC transplantation and traditional rehabilitation therapy	Seven of 10 subjects that received stem cells showed a significant enhancement in motor functions, auto-self ability and in muscular tension. Five subjects treated with the rehabilitative therapy showed some improvement in the same functions, but they were not statistically significant.	No side effects were recorded.	[79]
NCT01873547	Different Efficacy Between Rehabilitation Therapy and Stem Cells Transplantation in Patients With SCI in China	Phase 3	300 patients (20–65 years)	UC-MSCs	Intrathecal injection	UC-MSC transplantation and traditional rehabilitation therapy	-	-	-
**Neuronal Stem Cells**									
NCT01321333	Study of Human Central Nervous System Stem Cells (HuCNS-SC) in Patients With Thoracic Spinal Cord Injury	Phase 1/2	12 patients (18–60 years)	Human CNS-SCs	Intramedullary transplantation	Human CNS-SC transplantation	-	-	-
NCT01725880	Long-Term Follow-Up of Transplanted Human Central Nervous System Stem Cells (HuCNS-SC) in Spinal Cord Trauma Subjects	-	12 patients (18–60 years)	Human CNS-SCs	Intramedullary transplantation	Human CNS-SC transplantation	-	-	-
NCT02163876	Study of Human Central Nervous System (CNS) Stem Cell Transplantation in Cervical Spinal Cord Injury	Phase 2	31 patients (18–60 years)	Human CNS-SCs	Intramedullary transplantation	Human CNS-SC transplantation	-	-	-
NCT02302157	Dose Escalation Study of AST-OPC1 in Spinal Cord Injury	Phase 1/2	25 patients (18–69 years)	AST-OPC1 (10 million cells; two doses)	-	AST-OPC1 transplantation	-	-	-

MSCs, Mesenchymal Stem Cells; BM-MSCs, Bone Marrow MSCs; BMPCs, Bone Marrow Progenitor Cells; AT-MSCs, Adipose Tissue-derived MSCs; CNS-SCs, Central nervous System Stem Cells; UC-MSCs, Umbilical Cord MSCs; AIS, Association Impairment Scale; SSEP, Somatosensory Evoked Potentials; MRI, Magnetic Resonance Image.

**Table 2:** Ongoing clinical trials of stem cell therapy in SCI (https://clinicaltrials.gov/). The table lists all the clinical trials active in recruitment phase or that will be important to understanding the efficacy and safety of stem cell therapy in the management of SCI.

Identifier	Study Title	Phase	Subjects	Cell Therapy	Route of Administration	Intervention
Umbilical Cord Mesenchymal Stem Cells						
NCT03505034	Intrathecal Transplantation of UC-MSC in Patients With Late Stage of Chronic Spinal Cord Injury	Phase 2	30 patients (18–65 years)	UC-MSCs (1 × 10^6^ cells/kg; once a month for 4 times)	Intrathecal injection	Allogenic UC-MSC transplantation
NCT03521336	Intrathecal Transplantation of UC-MSC in Patients With Sub-Acute Spinal Cord Injury	Phase 2	84 patients (18–65 years)	UC-MSCs (1 × 10^6^ cells/kg; once a month for 4 times)	Intrathecal injection	Allogenic UC-MSC transplantation
NCT03521323	Intrathecal Transplantation of UC-MSC in Patients With Early Stage of Chronic Spinal Cord Injury	Phase 2	66 patients (≥18 years)	UC-MSCs (1 × 10^6^ cells/kg; once a month for 4 times)	Intrathecal injection	Allogenic UC-MSC transplantation
NCT03003364	Intrathecal Administration of Expanded Wharton’s Jelly Mesenchymal Stem Cells in Chronic Traumatic Spinal Cord Injury	Phase 1/2 a	10 patients (18–65 years)	WJ-MSCs (2 doses)	Intrathecal injection	WJ-MSC transplantation
**Adipose Tissue-derived Mesenchymal Stem Cells**						
NCT03308565	Adipose Stem Cells for Traumatic Spinal Cord Injury	Phase 1	10 patients (≥18 years)	AT-MSCs (100 million cells; a single dose)	Intrathecal injection	Autologous AT-MSC transplantation
**Bone Marrow Mesenchymal Stem Cells**						
NCT02574572	Autologous Mesenchymal Stem Cells Transplantation in Cervical Chronic and Complete Spinal Cord Injury	Phase 1	10 patients (18–65 years)	BM-MSCs (2 doses)	Percutaneous injection	Autologous BM-MSC transplantation
NCT02574585	Autologous Mesenchymal Stem Cells Transplantation in Thoracolumbar Chronic and Complete Spinal Cord Injury Spinal Cord Injury	Phase 2	40 patients (18–65 years)	BM-MSCs (2 doses)	Percutaneous injection	Autologous BM-MSC transplantation
NCT01676441	Safety and Efficacy of Autologous Mesenchymal Stem Cells in Chronic Spinal Cord Injury	Phase 2/3	32 patients (16–65 years)	BM-MSCs (1 × 10^6^ and 1 × 10^7^; two doses)	Intrathecal injection	Autologous BM-MSC transplantation
NCT02687672	Transplantation of Autologous Bone Marrow or Leukapheresis-Derived Stem Cells for Treatment of Spinal Cord Injury	Phase 1/2	50 patients (5–50 years)	BM-MSCs	-	Autologous BM-MSC transplantation
**Neuronal Stem Cells**						
NCT01772810	Safety Study of Human Spinal Cord-derived Neural Stem Cell Transplantation for the Treatment of Chronic SCI	Phase 1	8 patients (18–65 years)	Human Spinal Cord-derived Neural Stem Cells	-	Human Spinal Cord-derived Neural Stem Cell Transplantation
NCT04205019	Safety Stem Cells in Spinal Cord Injury	Phase 1	10 patients (18–40 years	Neuro-Cells	Intrathecal injection	Neuro-Cells Transplantation
NCT03935724	Clinical Study of an Autologous Stem Cell Product in Patients With a (Sub)Acute Spinal Cord Injury	Phase 2/3	8 patients (18–65 years	Neuro-Cells	-	Neuro-Cells Transplantation

MSCs, Mesenchymal Stem Cells; UC-MSCs, Umbilical Cord MSCs; WJ, Wharton’s Jelly MSCs; BM-MSCs, Bone Marrow MSCs; AT-MSCs, Adipose Tissue-derived MSCs.

## Abbreviations

SCI	Spinal Cord Injury
MSCs	Mesenchymal Stem Cells
TNF-α	Tumor Necrosis Factor-alpha
IL-1β	Interleukin 1-beta
IL-6	Interleukin-6
ROS	Reactive Oxygen Species
RNS	Reactive Nitrogen Species
MPSS	Methylprednisolone Sodium Succinate
BM-MSCs	Bone Marrow MSCs
AT-MSCs	Adipose Tissue-derived MSCs
UC-MSCs	Umbilical Cord MSCs
BDNF	Brain-Derived Neurotrophic Factor
GDNF	Glial cell-Derived Neurotrophic Factor
IGF-1	Insulin Grow Factors-1
OPCs	Oligodendrocytes Progenitor Cells
hESC	Human Embryonic Stem Cell
CNS	Central Nervous System
VEGF	Vascular Endothelial Growth Factor
NGF	Nerve Growth Factor
NT-3	Neurotrophin-3
FGF	Fibroblast Growth Factor
EGF	Epidermal Growth Factor
TGF-β	Transforming Growth Factor-beta
WJ-MSCs	Wharton’s Jelly MSCs
ASIA	American Spinal Injury Association
AIS	Association Impairment Scale
MRI	Magnetic Resonance Image
SSEP	Somatosensory Evoked Potentials
IANR-SCIFRS	International Association of Neurorestoratology Spinal Cord Injury Functional Rating Scale
BMPCs	Bone Marrow Progenitor Cells
CNS-SCs	CNS Stem Cells
CT	Computerized Tomography
